# Establishment and verification of a prognostic model based on coagulation and fibrinolysis-related genes in hepatocellular carcinoma

**DOI:** 10.18632/aging.205699

**Published:** 2024-04-01

**Authors:** Meng Fan, Le Lu, Hao Shang, Yuxuan Lu, Yi Yang, Xiuyan Wang, Hongwei Lu

**Affiliations:** 1 Department of General Surgery, The Second Affiliated Hospital of Xi’an Jiaotong University, Xi’an 710003, China; 2 Department of Medical, Shenzhen Engineering Center for Translational Medicine of Precision Cancer Immunodiagnosis and Therapy, YuceBio Technology Co., Ltd., Shenzhen 518038, China

**Keywords:** hepatocellular carcinoma, coagulation and fibrinolysis related genes, RiskScore model, prognosis, survival probability

## Abstract

Background: Studies have shown that coagulation and fibrinolysis (CFR) are correlated with Hepatocellular carcinoma (HCC) progression and prognosis. We aim to build a model based on CFR-correlated genes for risk assessment and prediction of HCC patient.

Methods: HCC samples were selected from The Cancer Genome Atlas (TCGA) and Gene Expression Omnibus (GEO) databases respectively. The Molecular Signatures Database (MSigDB) was used to select the CFR genes. RiskScore model were established by single sample gene set enrichment analysis (ssGSEA), weighted correlation network analysis (WGCNA), multivariate Cox regression analysis, LASSO regression analysis.

Results: PCDH17, PGF, PDE2A, FAM110D, FSCN1, FBLN5 were selected as the key genes and designed a RiskScore model. Those key genes were Differential expressions in HCC cell and patients. Overexpression PDE2A inhibited HCC cell migration and invasion. The higher the RiskScore, the lower the probability of survival. The model has high AUC values in the first, third and fifth year prediction curves, indicating that the model has strong prediction performance. The difference analysis of clinicopathological features found that a great proportion of high clinicopathological grade samples showed higher RiskScore. RiskScore were positively correlated with immune scores and TIDE scores. High levels of immune checkpoints and immunomodulators were observed in high RiskScore group. High RiskScore groups may benefit greatly from taking traditional chemotherapy drugs.

Conclusions: We screened CFR related genes to design a RiskScore model, which could accurately evaluate the prognosis and survival status of HCC patients, providing certain value for optimizing the clinical treatment of cancer in the future.

## INTRODUCTION

HCC is a serious threat to human health with strong invasion and high mortality [1]. In 2020, an estimated 9.06 million new cases of primary liver cancer were diagnosed all over the world, and about 8.3 million people died from liver cancer. The occurrence and death rate of liver cancer have been increasing annually [2]. Patients with cirrhosis have a very high likelihood of converting to liver cancer, and imaging and ultrasound monitoring are often used in early diagnosis of tumor when treatment is most effective [3]. Due to the allocation of medical resources and the latent nature of early liver cancer, most patients are diagnosed in the middle and late stages, which will increase the cost and risk of treatment [4, 5]. Despite advances in the treatment of liver cancer [6], most patients with HCC do not respond well to cell programmed death 1 (anti-PD1) therapy [7]. Therefore, the identification of key molecular features [8] with advanced techniques [9, 10] related to HCC is helpful for the specific mechanism research of HCC, and may provide new ideas for the search for more potential therapeutic targets and prognostic diagnosis in the future.

In recent years, the interaction between the growth of malignant tumor cells and the coagulation and fibrinolytic (CFR) systems has attracted much attention. Experiments have shown that targeting CFR systems may promote the course of malignant disease [11]. Cancers such as HCC are more likely to cause the formation of a variety of blood clots, including portal vein thrombosis [12]. Anticoagulant therapy, such as the application of heparin, may help us with antitumor therapy and can be involved in the intervention of many cancers and other diseases [13]. The tumor microenvironment (TME) and the coagulation cascade are related to malignancies [14]. The TME is a key mediator of cancer progression and treatment outcome, and is closely related to the outcome of immunotherapy [15].

HCC studies have found a significant correlation between coagulation-related genes and TME, and the coagulation-related genes have the potential as prognostic biomarkers [16]. Plasma fibrinogen is associated with the development and prognosis of a variety of cancers and can be used as a prognostic biomarker of some cancers [17]. Hyun Kyung Kim et al. found higher D-dimer and plasma fibrinogen levels in patients with advanced HCC, as well as a similarly high level of tumor thrombosis, suggesting that CFR continues to occur during tumor progression [18]. After HCC resection, there was also a significant increase in levels of the plasminin-alpha-2-plasminin-inhibitor complex [19]. Fibrinogen-like-protein 1 (FGL1) belongs to a relatively common branch of the fibrinogen-related protein (FREP) family. High level of FGL1 expression is associated with the development and prognosis HCC and is a potential prognostic biomarker [20]. Coagulation-related genes and fibrinolytic systems are associated with the development and prognostic effects of HCC.

At present, the relationship between CFR related genes and HCC development and prognosis remains unclear. In this study, we will enrich and screen CFR related genes based on multiple data sets to design a RiskScore model and test the predictive function of the model. Next, we will analyze the correlation between RiskScore and clinical indicators. We hope that this model can provide a new reference for the study of the pathogenesis of HCC and the optimization of clinical treatment plan.

## MATERIALS AND METHODS

### Data acquisition and preprocessing

(1) RNA-Seq data of TCGA-HCC were acquired from The Cancer Genome Atlas (TCGA) database using the GDC API [21]. 371 HCC samples and 52 para-cancer tissue samples were acquired. HCC samples were retained according to the clinicopathological information table, and samples with no survival state and time were removed, then log2 (TPM+1) was used in calculating the mRNA expression level. A total of 355 samples were screened. Detailed clinicopathological information was listed in Table 1.

**Table 1:** Detailed clinicopathological information for enrolled TCGA-HCC and GSE14520 samples.

**Characteristics**	**TCGA (*N* = 355)**	**GSE14520 (*N* = 221)**
**Status**		
Alive	226 (63.66%)	136 (61.54%)
Dead	129 (36.34%)	85 (38.46%)
**OS.time**		
Mean ± SD	821.69 ± 731.02	1213.40 ± 647.40
Median (min-max)	601.00 (1.00, 3675.00)	1569.00 (60.00, 2022.00)
**Age**		
Mean ± SD	59.81 ± 13.10	50.82 ± 10.62
Median (min-max)	61.00 (16.00, 90.00)	50.00 (21.00, 77.00)
**Gender**		
Female	115 (32.39%)	30 (13.57%)
Male	240 (67.61%)	191 (86.43%)
**Stage**		
I	166 (46.76%)	93 (42.08%)
II	80 (22.54%)	77 (34.84%)
III	82 (23.10%)	49 (22.17%)
IV	3 (0.85%)	
Unknown	24 (6.76%)	2 (0.90%)
**T.stage**		
T1	175 (49.30%)	
T2	87 (24.51%)	
T3	77 (21.69%)	
T4	13 (3.66%)	
Unknown	3 (0.85%)	
**N.stage**		
N0	241 (67.89%)	
N1	3 (0.85%)	
Unknown	111 (31.27%)	
**M.stage**		
M0	256 (72.11%)	
M1	3 (0.85%)	
Unknown	96 (27.04%)	
**Grade**		
G1	53 (14.93%)	
G2	169 (47.61%)	
G3	117 (32.96%)	
G4	11 (3.10%)	
Unknown	5 (1.41%)	

(2) GSE14520 expression data of HCC including 221 tumor samples were acquired from The Gene Expression Omnibus (GEO) database [22]. The GEO data was preprocessed as follows: We obtained the annotation data contained in the chip platform, and mapped the probe to the gene according to the annotation information to obtain the required expression level of each gene, then log2 (TPM+1) was used in calculating the mRNA expression level. A total of 221 samples were picked out. Detailed clinicopathological information was listed in Table 1.

(3) A total of 149 CFR-correlated genes were collected from the MSigDB [23].

### Screening and enrichment of differential expressed genes (DEGs)

We used limma packages to screen for DEGs between HCC and normal samples (*P* < 0.05) [24]. We used the ggplot2 package to make the volcano plot of DEGs [25]. Finally, we chose the clusterProfiler package for gene ontology (GO) and Kyoto Encyclopedia of Genes and Genomes (KEGG) enrichment analysis [26].

### WGCNA

WGCNA was used to select the gene modules most associated with CFR scores [27]. Firstly, the half of genes with the smaller mean absolute deviation [4] in the gene expression profile were excluded. The soft threshold (β) is obtained from the pickSoftThreshold() function included in WGCNA. Next, the gene modules were judged and divided by hierarchical clustering method, and the criteria of at least 50 genes per module (minModuleSize = 50) was used to find gene modules. Then we look for associations between modules and sample properties. Finally, we obtained the genes in the module showing the highest correlation and defined them as CFR related genes.

### Screening of key genes

DEGs was intersected with the module with the highest correlation, and the significant prognostic genes were acquired by univariate Cox regression analysis using coxph function of survival package (*P* < 0.05) [28]. The LASSO regression algorithm and randomForest algorithm were used to precisely narrow down the range of significant prognostic genes [29, 30]. The intersection of genes obtained by LASSO and randomForest algorithm was employed to reduce genes in the model by stepwise regression. Finally, we obtained the key genes that influence prognosis, and drew the multivariate forest plot of the key genes.

### Establishment and verification of RiskScore model

The TCGA-HCC dataset was used as a training set, and the GSE14520 dataset was a verification set. RiskScore is calculated as follows:


RiskScore=∑βi×Expi

Where i refers to the expression level of key genes, and β is the Cox regression coefficient of key genes.

Next, we calculated RiskScore for each sample of TCGA-HCC dataset. Patients are differentiated into high RiskScore and low RiskScore groups based on the most appropriate RiskScore threshold obtained from the survminer package. We used timeROC package for ROC analysis to analyze the prognostic efficiency of 1, 3 and 5 years. The AUC value of ROC curve was obtained and analyzed to judge the accuracy of the RiskScore model. Kaplan-Meier curve was made to analyze the prognostic survival chance. To better verify the model robustness, the GSE14520 dataset was validated using the same method.

### Analysis of clinicopathological features and construction of a nomogram

We drew a clustering heat map to compare the differences in clinicopathological features between RiskScore groups in TCGA-HCC dataset. Violin plots were also drawn to analyse the differences in clinicopathological grades between RiskScore groups. Univariate and multivariate Cox regression analysis was performed for Riskscore and clinicopathological features, and independent prognostic factors were selected. To assess the survival risk of patients, we combined RiskScore and independent prognostic factors to establish a nomogram through the rms package. Calibration curve is used to evaluate the prediction accuracy of the model. Besides, ggDCA package was used to make Decision curve (DCA) to analyse the reliability of the model [31]. Finally, ROC curve of various clinicopathological features on overall survival (OS) in 1-5 years was drawn [32].

### Gene set enrichment analysis (GSEA)

CFR score of TCGA-HCC samples and normal samples was acquired by ssGSEA [33]. The expression profiles of TCGA data sets were processed by Gene Set Variation Analysis (GSVA), and the score of each sample in the HALLMARK Pathway was calculated [34]. The correlation between RiskScore and channel score was calculated. We analyzed the differentially activated pathways, and GSEA was performed using theh.all.v7.5.1.symbols.gmt gene set in the MSigDB database [35]. FDR < 0.05 is a significant enrichment. Mutated data set of TCGA processed by mutect2 software was downloaded. We used Fisher’s exact test to acquire genes with high-frequency mutations in different groups, and drew a waterfall map.

### Immune microenvironment and drug sensitivity analysis

The relative abundance of 22 kinds of immune cells was determined by CIBERSORT package [36]. Six immune cell scores were calculated by TIMER [37]. We have obtained immunomodulators related to a variety of different immune processes (antigen presentation, cell adhesion, co-inhibitors, co-stimulants, ligands, receptors, and others) from existing studies [38]. The potential clinical effects of immunotherapy across RiskScore groups were analyzed using Tumor Immune Dysfunction and Exclusion (TIDE) method [39]. Finally, the sensitivity of different RiskScore groups to conventional chemotherapy drugs was calculated using pRRophetic package [40].

### Cell culture and transfection

HCC cell lines (HUH7) and Normal liver cell line (L0-2) were bought from the American Type Culture Collection (ATCC) cell bank. Cells were cultured in DMEM culture medium (Gibco, USA), supplemented with 10% FBS under a standard humidified incubator (5% CO_2_ at 37°C). The negative control (oe NC) and oe CEMIP (Sagon, China) were sub-cloned into the pCDNA3.1 expression vector (Invitrogen, Carlsbad, USA) and transfected into the cells by Lipofectamine 3000 reagent (Invitrogen, MA, USA).

### Quantitative real-time polymerase chain reaction (qRT-PCR)

Total RNA was extracted and reverse transcribed (Takara Bio, China). The expressions of different genes were detected by the PowerUp SYBR Master Mix Applied Biosystems (Invitrogen, USA). The sequences of primer pairs for the genes targeted were listed in Table 2. Gene mRNA levels were normalized to GAPDH mRNA levels with the formula: 2-(^CT target-CT GAPDH^), where CT is the threshold cycle.

**Table 2:** The sequences of primer pairs for target genes.

**Gene**	**Forward primer sequence (5′–3′)**	**Reverse primer sequence (5′–3′)**
PCDH17	AGCCAACCACTTGAACAAGAACC	GCATCCAGCACCTGTCAGAATG
PGF	CGGCTCGTCAGAGGTGGAAG	CAGCAGGGAGACACAGGATGG
PDE2A	CTGTCTACACCTACCTACTGGATGG	AGCCGCTTCTGGGAGATGATAG
FAM110D	GAACAAAGAGAACGCCAAG	GAACAAAGAGAACGCCAAG
FSCN1	CAGGTCAACATCTACAGCGTCAC	CTGGAAGGCGAGGGTGATGAG
FBLN5	GCCAGTCAGGACAGTGTTTAGATG	TGAGGCAGAAGAATCGCTTGAAC
GAPDH	CTCGCTTCGGCAGCACA	AACGCTTCACGAATTTGCGT

### Migration and invasion assays

Migration and invasion experiments were carried out three times as described previously [41]. Briefly, cell invasion was tested employing Matrigel-coated transwell inserts (Transwell, Corning Costar) with a total of 4 × 10^4^ cells were added to the upper chamber. After incubation at 37°C for 24 hours, the non-invading cells were removed, and invaded cells were counted in five random fields with a microscope. The procedure for cell migration is similar to that of invasion, except for no matrigel.

### Immunohistochemistry (IHC)

The medical approval of this study was received from the Ethics Committee of The Second Affiliated Hospital of Xi’an Jiaotong University. IHC validation of key gene expressions was performed in tissues of HCC patients. Tissue samples were soaked in a dewaxing solution and 95% ethanol for dewaxing, then heated in the microwave to recover the antigen. After being blocked with 5% goat serum, Anti-FAM110D (ab234868, 1:500, Abcam, USA), anti-FBLN5 (ab66339, 1:500, Abcam), anti-FSCN1 (ab126772, 1:500, Abcam), Anti-pcdh17 (ab128815, 1:500, Abcam) Anti-PDE2A (ab224616, 1:500, Abcam) Anti-PGF (10642-1-AP, 1:500, Proteintech, China) were used for IHC analysis. Tissue samples were incubated with antibodies at 4°C for 8 h and then co-incubated secondary antibodies at room temperature for 60 min. The immunoreactivity was observed after incubation with diaminobenzidine for 5 minutes. The stained tissue was observed under a microscope.

### Statistical analysis

This study mainly uses R software for statistical analysis. The comparison between the different groups was analyzed by Wilcoxon test. The Spearman method was used to calculate correlation. The *P*-values with significant differences were all less than 0.05.

### Availability of data and materials

The datasets generated and/or analyzed during the current study are available in the (GSE14520) repository, (https://www.ncbi.nlm.nih.gov/geo/query/acc.cgi?acc=GSE14520).

## RESULTS

### Screening and enrichment of DEGs

**Figure 1:**
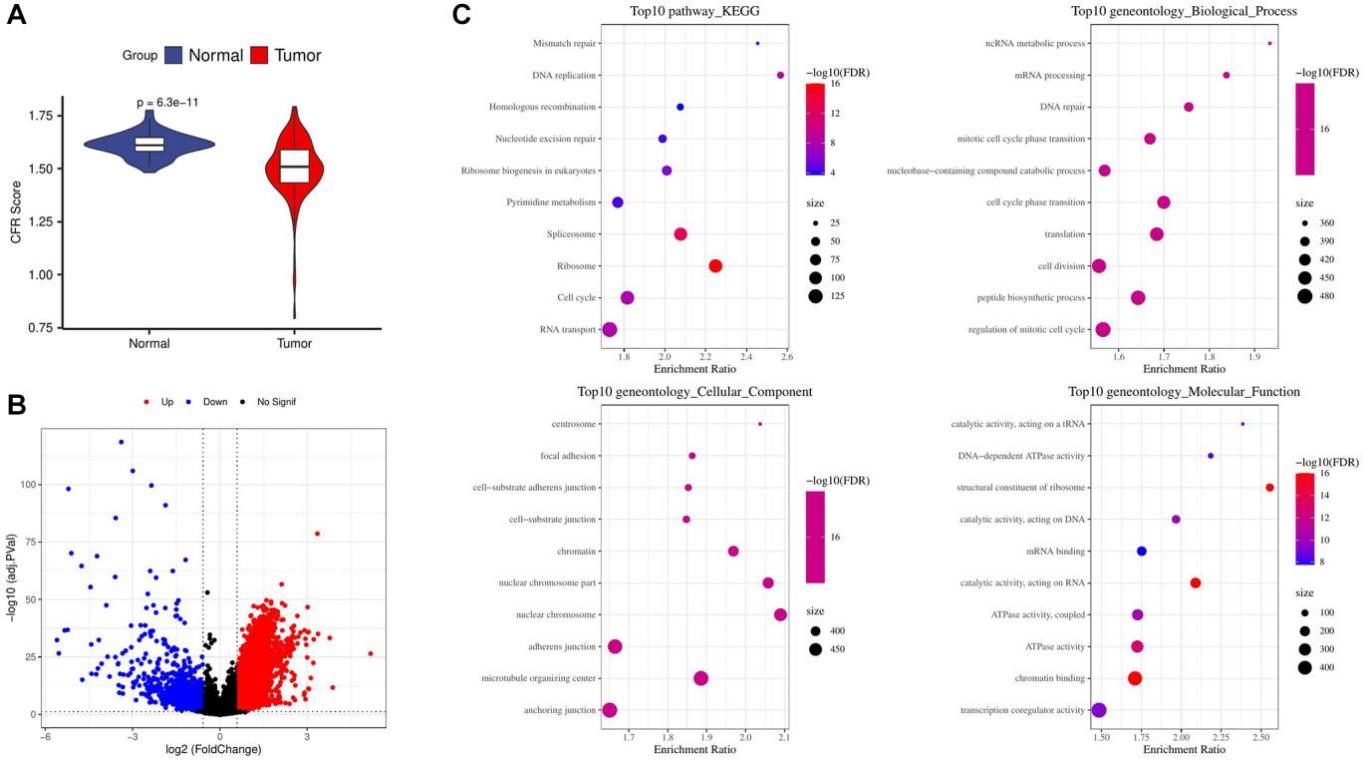
**Screening and enrichment of DEGs.** (**A**) Difference in CFR score between tumor and normal control samples in the TCGA cohort; (**B**) Volcano plot for screening differential expressed genes in the TCGA cohort; (**C**) Enrichment of tumor up-regulated genes by GO and KEGG analysis.

The TCGA-HCC sample had a lower CFR score (Figure 1A). 6724 up-regulated genes and 822 down-regulated genes were obtained by screening the DEGs between TCGA-HCC samples and normal samples (Figure 1B).

We used GO and KEGG enrichment analysis of genes up-regulated and found that the results were significantly enriched in cell cycle related pathways and biological processes. According to the enrichment results of top 10 KEGG pathways, the DNA replication pathway was the most significant. From the results of cellular component, nuclear chromosome part and nuclear chromosome are the most significant components. In terms of biological process results, ncRNA metabolic process and mRNA processing were the most significant biological processes. From the results of molecular function, structural constituent of ribosome was the most significant function (Figure 1C).

### WGCNA identifies genes associated with CFR score

**Figure 2:**
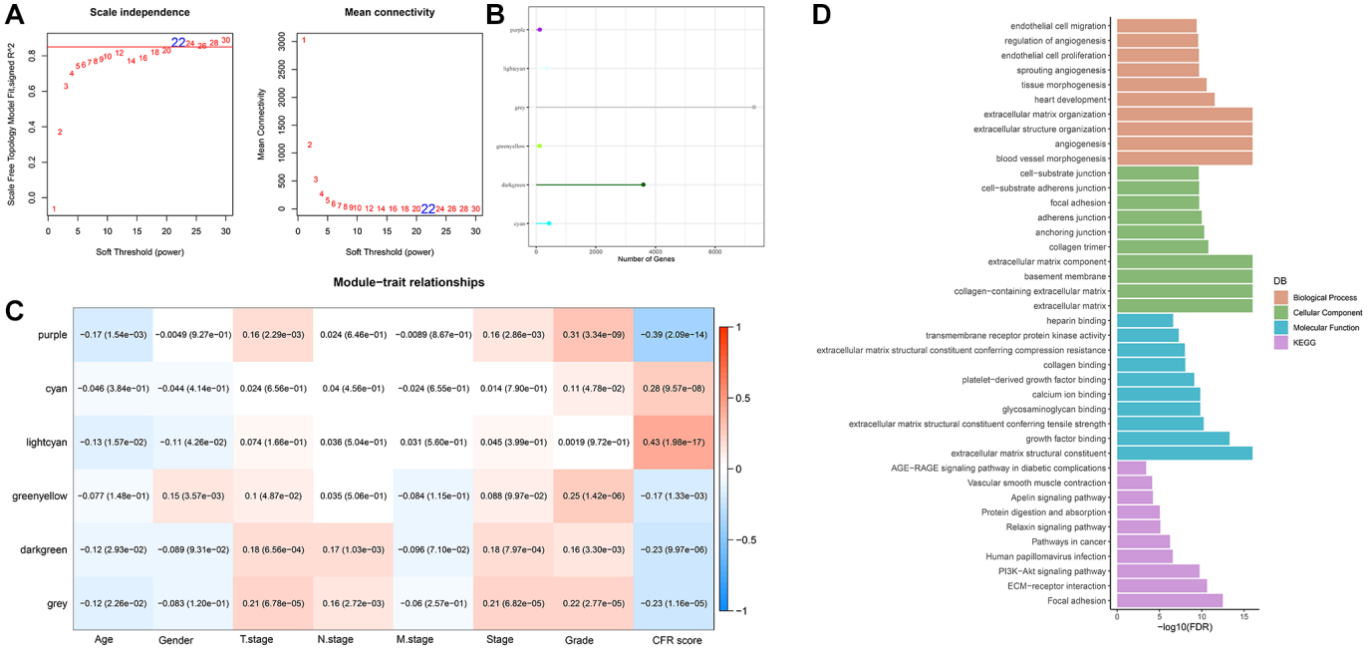
**WGCNA identifies genes associated with CFR score.** (**A**) Selection of soft threshold (β); (**B**) The number of module genes; (**C**) Correlation between feature vectors of modules and clinicopathological information; (**D**) Gene functional enrichment analysis of light cyan module.

The samples were clustered to screen co-expression modules. When the soft threshold β = 22 is selected, the network is scale-free (Figure 2A). Next, hierarchical clustering was used to divide gene modules. Six co-expression modules were generated after the combination of modules, among which grey module contains genes that cannot be classified into other modules. The number of genes in each module was shown in Figure 2B. Comparison of correlation between each module and clinicopathological features showed that lightcyan module had a positive correlation with CFR score (Figure 2C). Next, we conducted gene enrichment analysis in lightcyan module, and these genes were significantly enriched in biological processes related to angiogenesis (Figure 2D).

### Screening of key genes

**Figure 3:**
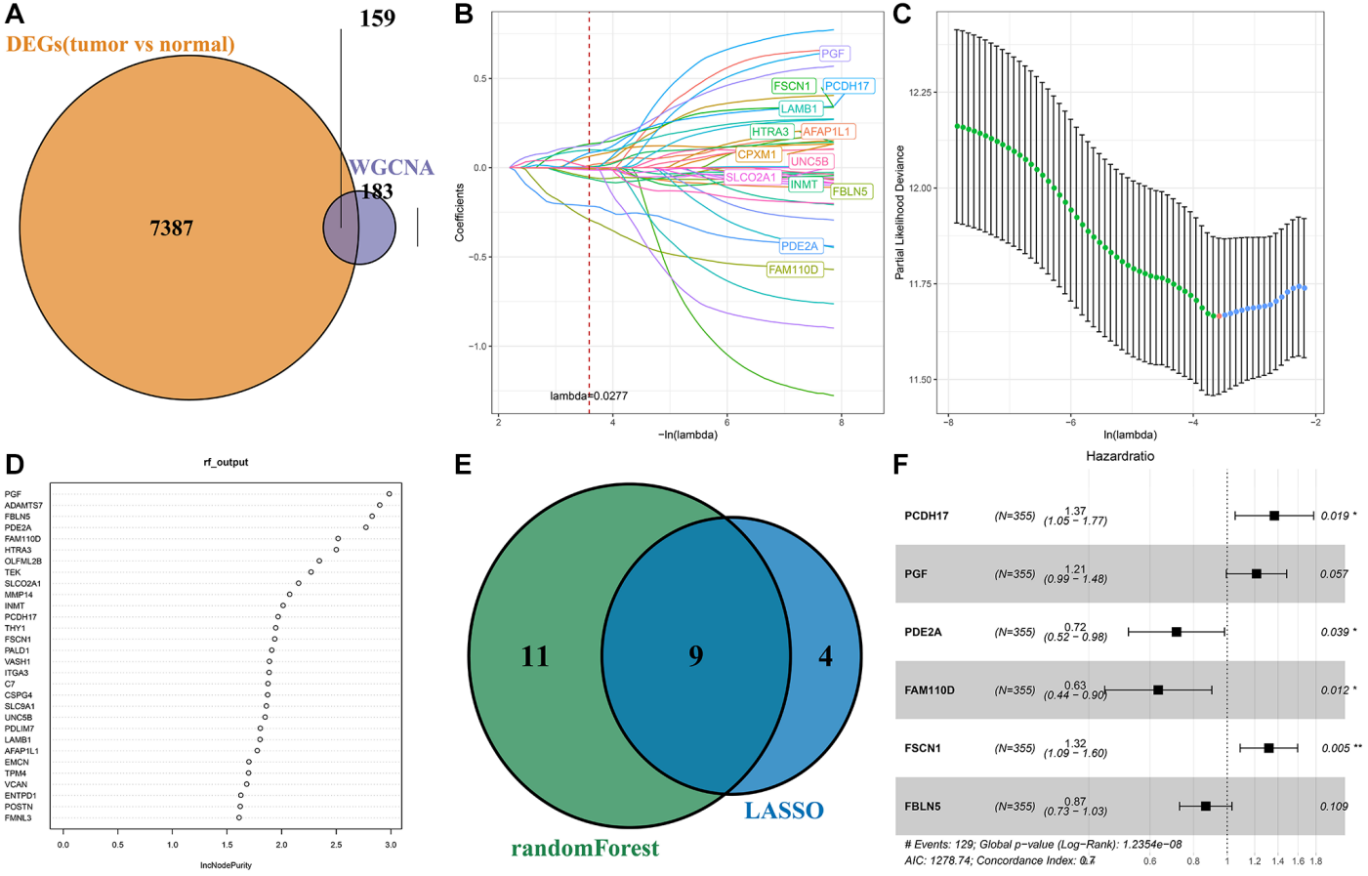
**Screening of key genes.** (**A**) Intersection of differential expressed genes and WGCNA screened module genes; (**B**) The trajectory of each independent variable; (**C**) Confidence interval for each lambda; (**D**) Sequencing of genes screened by randomForest algorithm; (**E**) The result intersection of LASSO algorithm and randomForest algorithm; (**F**) Multivariate forest map of key genes.

By intersecting the DEGs and lightcyan modules, 159 genes were obtained (Figure 3A). Univariate Cox analysis of 159 genes showed that 41 genes with greater prognostic influence were identified by consensus (*P* < 0.05). According to LASSO analysis, the number of independent variable coefficients approaching 0 rose together with the progressive increase in lambda (Figure 3B). The model was built using 10-fold cross-validation, which was then used to examine confidence intervals. We chose 13 genes as candidate genes at lambda = 0.0277, as this was when the model was the most accurate (Figure 3C). According to randomForest algorithm screening, we obtained the importance of genes and ranked the top 20 genes for further analysis (Figure 3D). The genes obtained by the two algorithms were intersected, and there were 9 overlapping genes (Figure 3E). Then the range of genes is compressed by stepwise regression. Finally, 6 genes were acquired as key genes affecting prognosis, including PCDH17, PGF, PDE2A, FAM110D, FSCN1 and FBLN5. Subsequently, multivariate forest map of key genes was drawn (Figure 3F).

### Establishment and verification of RiskScore model

Finally, the RiskScore model formula is as follows:

RiskScore = 0.312 × PCDH17 + 0.194 × PGF+(−0.335 × PDE2A) + (−0.457 × FAM110D) + 0.276 × FSCN1 + (−0.142 × FBLN5).

**Figure 4:**
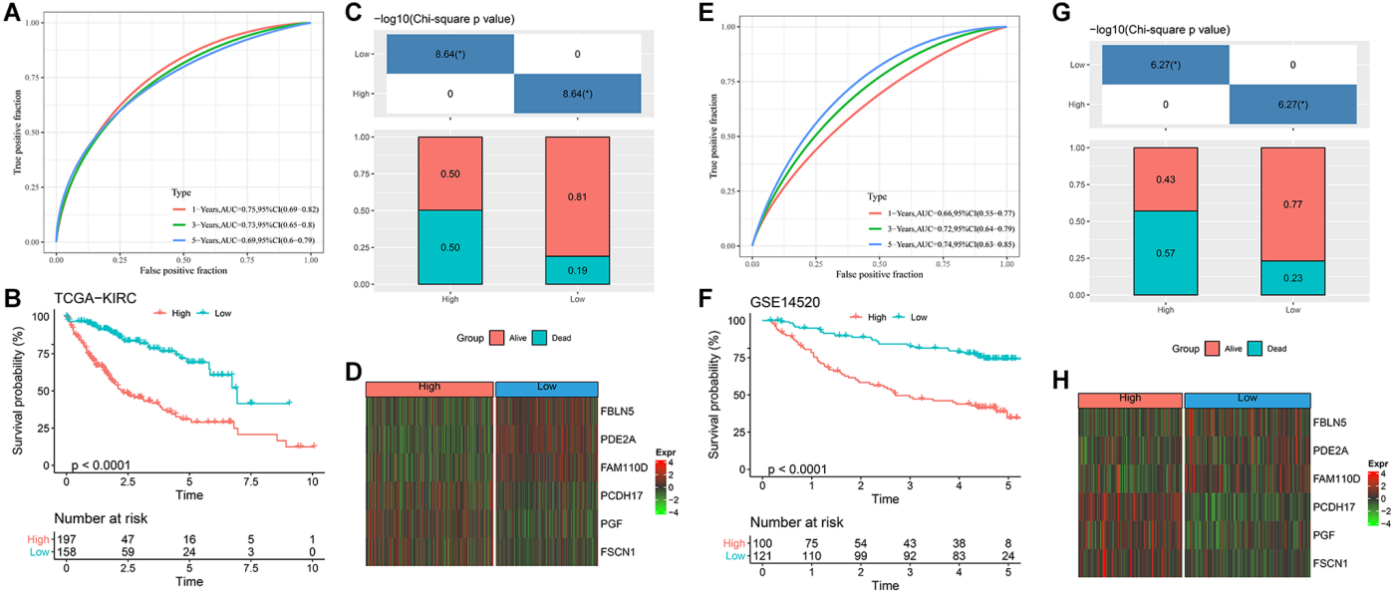
**Design and verification of RiskScore model.** (**A**) ROC curves for 1, 3 and 5 years of RiskScore model in TCGA-HCC; (**B**) KM curve of different RiskScore groups in TCGA-HCC; (**C**) Survival state between different RiskScore groups of TCGA-HCC; (**D**) Expression of key genes in TCGA-HCC; (**E**) ROC curves for 1, 3 and 5 years of RiskScore model in GSE14520 dataset; (**F**) KM curve of different RiskScore groups in GSE14520 dataset; (**G**) Survival state between different RiskScore groups of GSE14520 dataset; (**H**) Expression of key genes in GSE14520 dataset. ^*^p < 0.05.

After calculating the RiskScore of each sample in TCGA-HCC, they were divided into high and low RiskScore groups for ROC analysis of prognosis. The prognosis classification of prediction at 1, 3 and 5 years was analyzed respectively, and the results showed that the AUC at 1 year was 0.75, the AUC at 3 years was 0.73, and the AUC at 5 years was 0.69, indicating that the model had a high AUC value (Figure 4A). Meanwhile, the survival probability of the low RiskScore group was significantly better (*P* < 0.0001, Figure 4B, 4C). The expression of key genes in the high and low RiskScore groups showed that FBLN5, PDE2A and FAM110D were more prominent in the low RiskScore group, while PCDH17, PGF and FSCN1 were more prominent in the high RiskScore group (Figure 4D). We used the same method to verify the GSE14520 dataset, and the results showed that the AUC value was still high at 1, 3 and 5 years (Figure 4E). The survival probability of the low RiskScore group was significantly higher (Figure 4F, 4G). The expression level of key genes in GSE14520 dataset was similar to that of the TCGA-HCC dataset (Figure 4H). Therefore, the model has good predictive ability.

### Analysis of clinicopathological features and verification of a nomogram

**Figure 5:**
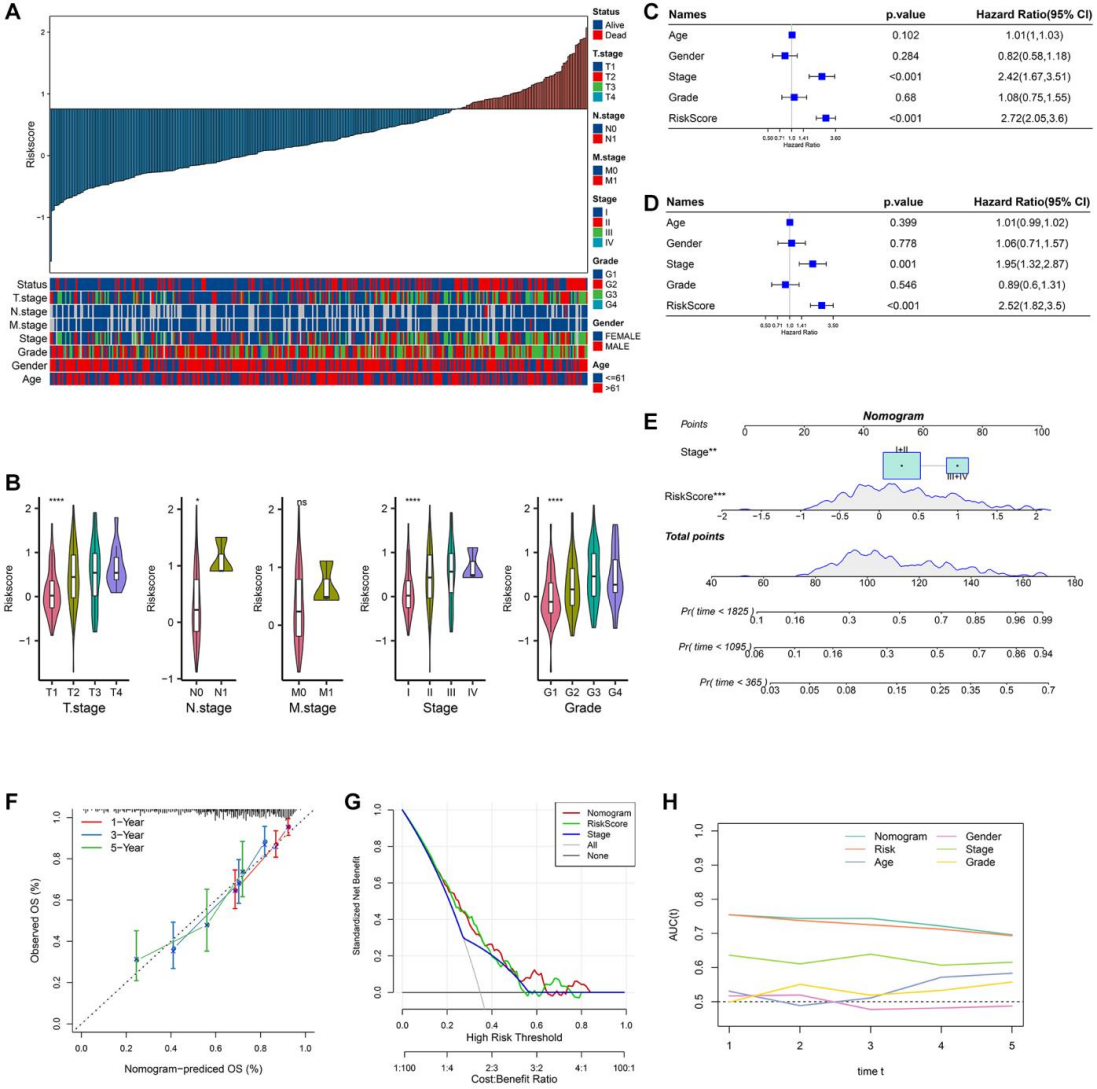
**Analysis of clinicopathological features and nomogram.** (**A**) RiskScore and distribution of clinicopathological features; (**B**) Analysis of different clinicopathological grades between different RiskScore groups; (**C**) Univariate Cox analysis of RiskScore and clinicopathological features; (**D**) Multivariate Cox analysis of clinicopathological features and RiskScore; (**E**) Construction of the nomogram; (**F**) 1, 3, 5 years calibration curve of the nomogram; (**G**) The decision curve of RiskScore, nomogram and clinicopathological features; (**H**) The ROC curves of a variety of clinicopathological features for overall survival (OS) at 1~5 years.

By comparing the clinicopathologic characteristics between the different RiskScore groups in the TCGA cohort, the higher the RiskScore, the larger the proportion of samples with high clinicopathological grade (Figure 5A). The differences in RiskScore among different clinicopathological grades, including T.stage, N.stage, M.stage, Stage and Grade, were also compared. The results showed that RiskScore also increased with the increase of each clinicopathological grade (Figure 5B). We also compared the prognostic differences between high and low risk groups in different Gender, Age Stages, and Grade groups in the TCGA dataset. We found that our risk group displayed significant difference in survival across different clinical groups, demonstrating that risk grouping has strong independence and is not easily influenced by other clinical factors (Supplementary Figure 1).

The *P*-values of RiskScore and Stage were both less than 0.001 in univariate Cox analysis (Figure 5C). The *P*-values of RiskScore and Stage were both less than 0.005 in the multivariate Cox analysis (Figure 5D). Stage and RiskScore were important prognostic variables. A nomogram was created using Stage, RiskScore, and Stage combined. The findings demonstrated that RiskScore had the greatest influence on the prediction of survival chances (Figure 5E). The fact that the 1, 3, and 5 year(s) of anticipated calibration points were near the standard curve indicated that the nomogram had a strong predictive potential (Figure 5F). DCA results show that RiskScore and nomogram have higher benefits than extremum curves, so they both have strong stability and prediction ability (Figure 5G). Compared with other clinicopathological features, both nomogram and RiskScore showed the better survival prediction ability (Figure 5H).

### Immunological abnormalities and drug susceptibility between RiskScore groups

**Figure 6:**
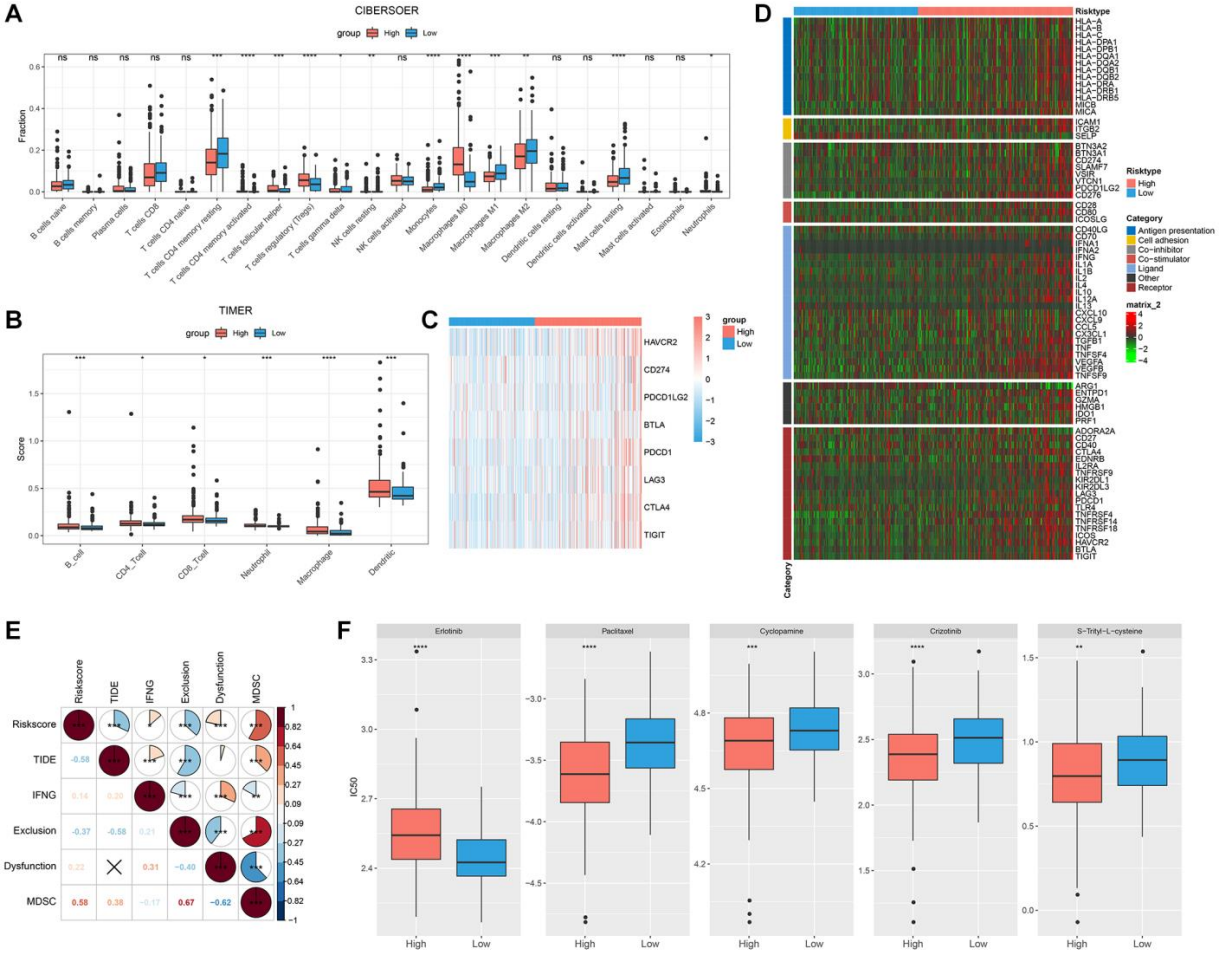
**Immunological abnormalities and drug susceptibility between RiskScore groups.** (**A**) Differences in immune infiltration by CIBERSORT algorithm; (**B**) Differences in immune score by TIMER; (**C**) Analysis of immune checkpoint gene expression; (**D**) Differences in the expression of immunomodulators; (**E**) Correlation between RiskScore and TIDE score; (**F**) Comparison of drug sensitivity between different RiskScore groups.

The relative abundance of 22 distinct immune cell types was calculated using the CIBERSORT method, and 12 immune cell types, such as T cells CD4 memory active and T cells CD4 memory resting, significantly differed across various RiskScore groups (Figure 6A). The immunity scores were calculated using the TIMER, and the high RiskScore group had a higher immunity score (Figure 6B). Common immune checkpoints in the high RiskScore group showed high expression (Figure 6C). We compared the expression of common immunomodulators in different RiskScore groups, and the high RiskScore group also showed high expression (Figure 6D). Finally, TIDE was used to analyze the potential clinical effects of immunotherapy between RiskScore groups, and we found that RiskScore and TIDE score showed a significant negative correlation, suggesting that immunotherapy may be favorable in the high RiskScore group (Figure 6E).

Additionally, we analyzed how various RiskScore groups responded to conventional chemotherapy medications. More patients in the low RiskScore category were susceptible to erlotinib. Five conventional chemotherapy treatments, namely, S-Trityl-L-cysteine, Cyclopamine, Paclitaxel, and Crizotinib, were more likely to be advantageous to the high RiskScore group (Figure 6F).

### Abnormal biological pathways and gene mutations between different RiskScore groups

**Figure 7:**
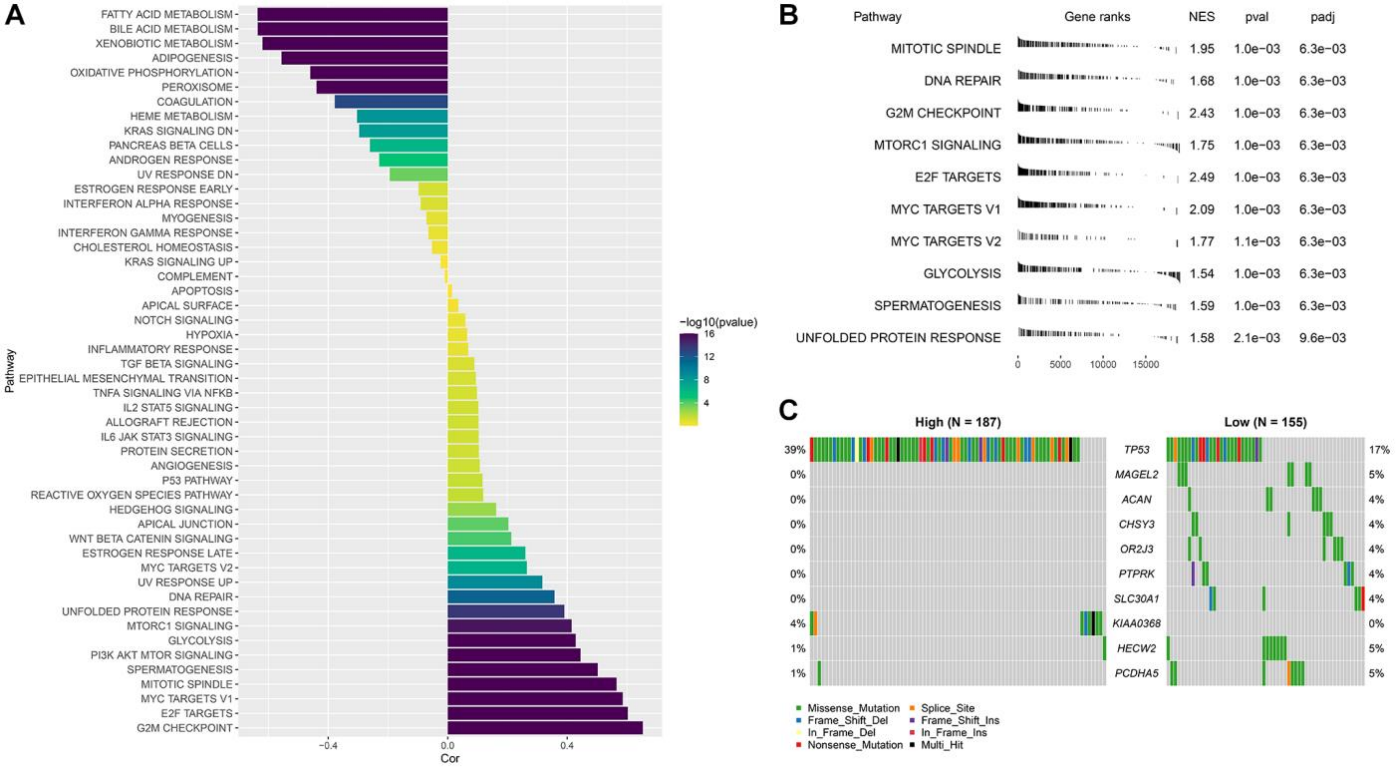
**Abnormal biological pathways and gene mutations between different RiskScore groups.** (**A**) Correlation between RiskScore and HALLMARK pathway score; (**B**) GSEA analysis between RiskScore groups; (**C**) Waterfall map of mutant gene differences between different RiskScore groups.

The correlation between RiskcSore and HALLMARK pathway score was calculated. RiskScore was positively correlated with cell cycle related pathways, and G2M CHECKPOINT was the most positively correlated pathway. RiskScore was negatively correlated with metabolism related pathways, and FATTY ACID METABOLISM was the most negatively correlated pathway (Figure 7A).

The high RiskScore group was mainly enriched in cell cycle related pathways, while the low RiskScore group had no significantly enriched pathways (Figure 7B). The analysis of the differences of mutant gene characteristics between different groups showed that the mutation frequency of TP53 was higher in the high RiskScore group, and more missense mutations occurred (Figure 7C).

### Validation the expression of model genes in HCC cell lines

**Figure 8:**
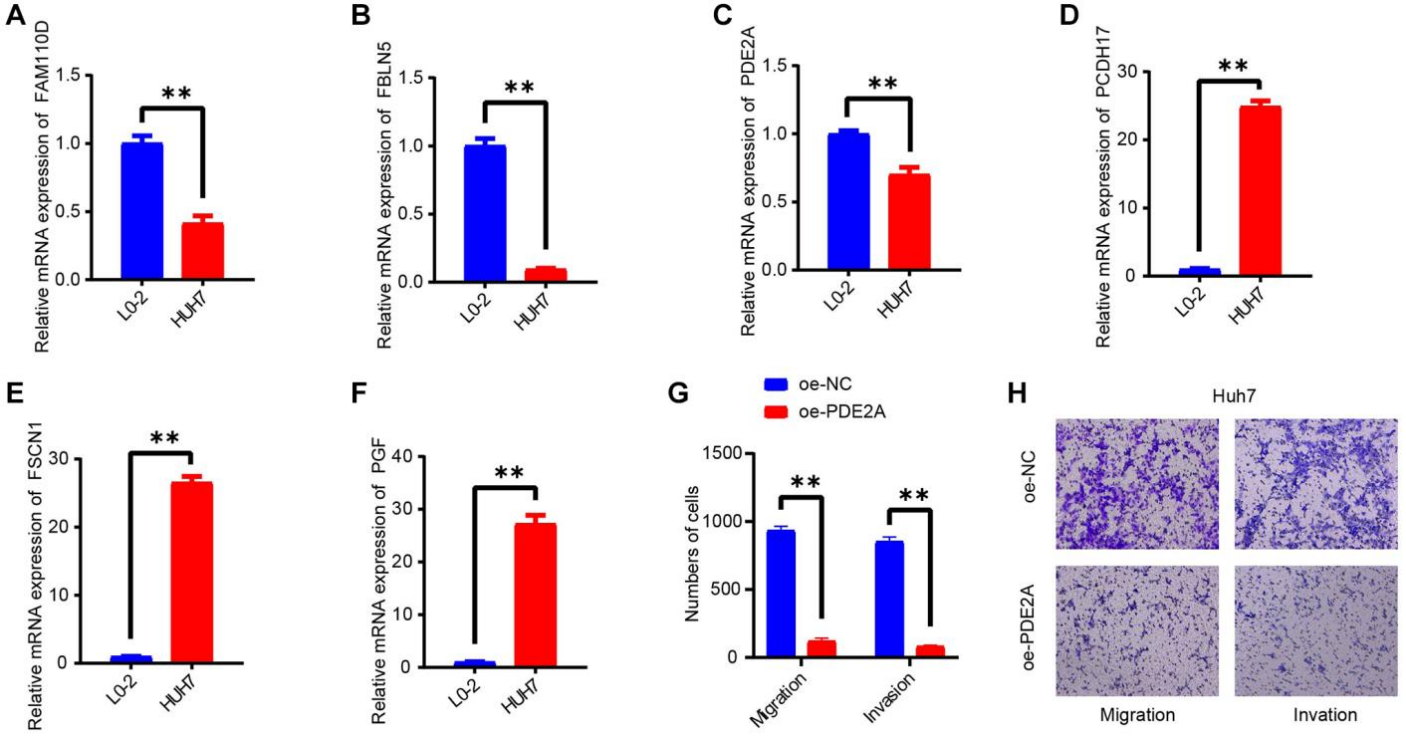
***In vitro* validating experiments.** (**A**–**F**) QRT-PCR detected the expressions of 6 model genes in human normal liver cells (L0-2) and HCC cell lines (HUH7). (**G**, **H**) Migration and invasion assays after PDE2A overexpression. *N* = 3, ^**^p < 0.01. The results are presented as mean ± SEM.

In this work, we selected 6 hub genes for RiskScore model construction. The expressions of these genes were validated in a vitro experiment. As seen in Figure 8A–8F, we could see that the levels of FAM110D, FBLN5 and PDE2A were all elevated in HUH7 cells compared with L0-2 cells (*p* < 0.01). While the levels of PCDH17, PGF and FSCN1 displayed opposite tendencies. IHC analysis also had similar results (Supplementary Figure 2).

### Overexpression of PDE2A depressed cell metastasis of HCC cells

As PDE2A was negatively correlated with tumor growth status, vascular infiltration, and promoted the HCC progression [42], and metastasis is one of the leading causes of cancer death [43]. Thereby, we detected the action of PDE2A on HCC development via cell migration and invasion assays. As displayed in Figure 8G, 8H, the number of migration and invasion cells were distinctly decreased after CEMIP overexpression. The results indicated that PDE2A could inhibit the metastasis of HCC cells.

## DISCUSSION

Abnormal expression of CFR related genes has been detected in a variety of cancers. Coagulation phenomena such as thromboembolism, including venous and arterial events, are frequently found in cancer patients and are directly or indirectly associated with cancer mortality, morbidity and the need for anticoagulant therapy [44]. Currently, the involvement of coagulation-related proteins in angiogenesis and cancer cell proliferation has been studied, and it has been found that inhibiting the clotting system may be helpful in reducing the development of tumors [45]. K-D Chen et al. found that tissue factor (TF) and coagulation factor VII (FVII) can jointly act on protease-activating receptor 2 (PAR2) to promote tumor growth, so coagulation factor can also accelerate tumor progression under certain conditions [46]. The Plasma TF levels in HCC patients affect tumor differentiation, proliferation and cancer progression to some extent [47]. After transcatheter hepatic artery embolization, thrombin-antithrombin III complex (TAT) levels in HCC patients were significantly increased, while antithrombin III and antiplasmin (α-2-plasmin inhibitor) levels were significantly decreased [48]. However, there are few studies on the association between HCC and CFR. Therefore, a RiskScore model was designed by combining the characteristics of CFR related genes in HCC to better assess the prognosis of patients.

In this study, six key genes, PCDH17, PGF, PDE2A, FAM110D, FSCN1 and FBLN5, were found to have a great influence on survival probability, and the model was built based on them. There have been studies showing strong links between these key genes and cancer. According to Yan Liu et al., a lower expression level of PCDH17 in HCC tissues may contribute to patients’ poor prognoses, while higher levels of PCDH17 expression can more effectively suppress HCC from growing and metastasizing [49]. We detected that PCDH17 was influenced by other genes in HCC. For example, Xiang, Y et al. found that MiR-23a-3p could facilitate G1/S cell cycle transition by inhibiting the expression of PCDH17 in HCC [50]. Also, PCDH17 is modulated by methylation of DNMT3B and had an impact on the malignant biological action on HCC [49]. Alpini et al. found that the expression level of PGF protein in HCC tissues was significantly higher, which may be helpful in inducing tumor angiogenesis, which may be related to the prognostic recurrence of HCC [51]. The expression of PDE2A was down-regulated in various cancer types including HCC. PDE2A was negatively correlated with tumor growth status, vascular infiltration, and promoted the HCC progression through modulating mitochondrial morphology and ATP content [42] as well influencing cell cycle of HCC [52]. In our cell experiment, we also found that elevated PDE2A could repress the metastasis of HCC cells. The immune function of HCC patients with low expression of PDE2A showed a downward trend [42]. There are few studies on FAM110D gene, but some studies have found that another FAM110 gene, FAD110B, can inhibit the growth and invasion of lung adenocarcinoma by inactivating the Wnt/β-catenin signaling pathway [53]. FAM110A may induce the occurrence and progression of pancreatic cancer [54]. Here the reduced levels of FAM110D in HCC cells may imply its anticancer effect. Fascin homologous 1 (FSCN1) was identified as a direct target of miR-539, a tumor suppressor, and its high expression level enhanced the aggressiveness of HCC cells [55]. Similarly, its high expression level was also observed in our validating experiment. Jia-Cheng Tang et al. found that FBLN-5 expression was down-regulated in HCC. FBLN-5 can inhibit tumor cell movement and proliferation through integrin-dependent mechanism, and its downregulation is significantly associated with advanced cancer cell metastasis [56]. Collectively, in this project, these genes were selected and validated as the key genes affecting prognosis.

According to the above results, the higher the RiskScore, the lower the survival probability, and the higher the proportion of samples with high clinicopathological grade. Moreover, in pathway analysis, RiskScore was positively correlated with the expression level of cell cycle related pathways, which might be related to abnormal regulation of cancer cell cycle [57]. It was found that G2M CHECKPOINT was the pathway with the strongest positive correlation, and FATTY ACID METABOLISM was the pathway with the strongest negative correlation. The alteration of lipid metabolism pathway may influence the occurrence, proliferation and spread of cancer [58]. The analysis of gene characteristics of gene mutation showed that patients with high RiskScore had a significantly increased probability of TP53 mutations. TP53, an important tumor suppressor gene, is frequently observed to have mutations in the genome of patients with various cancers. Mutation of TP53 will reduce its ability to inhibit tumor proliferation. And mutated p53 protein does not have normal biological function and may even induce tumor [59]. We also found significant increases in immune checkpoint and immunomodulator expression levels in the high RiskScore state. The reason may be that overexpression of immune checkpoint genes may hinder immune activation in HCC and promote the escape and spread of cancer cells [60].

Next, the limitations and development directions of this study will be elaborated. The collection amount of tumor sample data and gene sample data is small, and there is a lack of complete clinicopathological studies. More samples of clinicopathological data need to be integrated for later analysis. In addition, the specific role of CFR related genes in HCC and the pathogenesis of HCC have not been clarified, and further studies are still needed for HCC.

## CONCLUSIONS

In this study, CFR related genes were enriched and screened, and six key genes, namely PCDH17, PGF, PDE2A, FAM110D, FSCN1 and FBLN5, were obtained. The RiskScore model was constructed based on six key genes, which could effectively predict the survival of patients, and may provide new ideas for targeted therapy and specific mechanism research of HCC.

## Supplementary Materials

Supplementary Figures

## References

[1] Forner A, Llovet JM, Bruix J. Hepatocellular carcinoma. Lancet. 2012; 379:1245–55. 10.1016/S0140-6736(11)61347-0 22353262 PMC13537732

[2] Sung H, Ferlay J, Siegel RL, Laversanne M, Soerjomataram I, Jemal A, Bray F. Global Cancer Statistics 2020: GLOBOCAN Estimates of Incidence and Mortality Worldwide for 36 Cancers in 185 Countries. CA Cancer J Clin. 2021; 71:209–49. 10.3322/caac.21660 33538338

[3] Forner A, Reig M, Bruix J. Hepatocellular carcinoma. Lancet. 2018; 391:1301–14. 10.1016/S0140-6736(18)30010-2 29307467

[4] Yang JD, Hainaut P, Gores GJ, Amadou A, Plymoth A, Roberts LR. A global view of hepatocellular carcinoma: trends, risk, prevention and management. Nat Rev Gastroenterol Hepatol. 2019; 16:589–604. 10.1038/s41575-019-0186-y 31439937 PMC6813818

[5] Befeler AS, Di Bisceglie AM. Hepatocellular carcinoma: diagnosis and treatment. Gastroenterology. 2002; 122:1609–19. 10.1053/gast.2002.33411 12016426

[6] Yan D, Li C, Zhou Y, Yan X, Zhi W, Qian H, Han Y. Exploration of Combinational Therapeutic Strategies for HCC Based on TCGA HCC Database. Oncologie. 2022; 24:101–11. 10.32604/oncologie.2022.020357

[7] Wei CY, Zhu MX, Zhang PF, Huang XY, Wan JK, Yao XZ, Hu ZT, Chai XQ, Peng R, Yang X, Gao C, Gao J, Wang SW, et al. PKCα/ZFP64/CSF1 axis resets the tumor microenvironment and fuels anti-PD1 resistance in hepatocellular carcinoma. J Hepatol. 2022; 77:163–76. 10.1016/j.jhep.2022.02.019 35219791

[8] Wang L, Ma X, Chen Y, Zhang J, Zhang J, Wang W, Chen S. MiR-145-5p Suppresses Hepatocellular Carcinoma Progression by Targeting ABHD17C. Oncologie. 2022; 24:897–912. 10.32604/oncologie.2022.025693

[9] Wang X, Xu J, Gu Q, Tang D, Ji H, Ju S, Wang F, Chen L, Yuan R. A UHPLC/MS/MS Assay Based on an Isotope-Labeled Peptide for Sensitive miR-21 Detection in HCC Serum. Oncologie. 2022; 24:513–26. 10.32604/oncologie.2022.024373

[10] Li S, Wang P, Qiu J, Xie Y, Yin D, Deng K. Comparison of IDEAL-IQ and IVIM-DWI for Differentiating between Alpha Fetoprotein-Negative Hepatocellular Carcinoma and Focal Nodular Hyperplasia. Oncologie. 2022; 24:527–38. 10.32604/oncologie.2022.022815

[11] Zacharski LR, Wojtukiewicz MZ, Costantini V, Ornstein DL, Memoli VA. Pathways of coagulation/fibrinolysis activation in malignancy. Semin Thromb Hemost. 1992; 18:104–16. 10.1055/s-2007-1002415 1574711

[12] Faccia M, Santopaolo F, Gasbarrini A, Pompili M, Zocco MA, Ponziani FR. Risk factors for portal vein thrombosis or venous thromboembolism in a large cohort of hospitalized cirrhotic patients. Intern Emerg Med. 2022; 17:1327–34. 10.1007/s11739-022-02928-8 35076898 PMC9352602

[13] Korte W. Changes of the coagulation and fibrinolysis system in malignancy: their possible impact on future diagnostic and therapeutic procedures. Clin Chem Lab Med. 2000; 38:679–92. 10.1515/CCLM.2000.099 11071061

[14] Saidak Z, Soudet S, Lottin M, Salle V, Sevestre MA, Clatot F, Galmiche A. A pan-cancer analysis of the human tumor coagulome and its link to the tumor immune microenvironment. Cancer Immunol Immunother. 2021; 70:923–33. 10.1007/s00262-020-02739-w 33057845 PMC10991611

[15] Liu Y, Xun Z, Ma K, Liang S, Li X, Zhou S, Sun L, Liu Y, Du Y, Guo X, Cui T, Zhou H, Wang J, et al. Identification of a tumour immune barrier in the HCC microenvironment that determines the efficacy of immunotherapy. J Hepatol. 2023; 78:770–82. 10.1016/j.jhep.2023.01.011 36708811

[16] He Q, Yang J, Jin Y. Immune infiltration and clinical significance analyses of the coagulation-related genes in hepatocellular carcinoma. Brief Bioinform. 2022; 23:bbac291. 10.1093/bib/bbac291 35849048

[17] Takagi H, Manabe H, Kawai N, Goto S, Umemoto T. Plasma fibrinogen and D-dimer concentrations are associated with the presence of abdominal aortic aneurysm: a systematic review and meta-analysis. Eur J Vasc Endovasc Surg. 2009; 38:273–7. 10.1016/j.ejvs.2009.05.013 19560946

[18] Kim HK, Lee KR, Yang JH, Yoo SJ, Lee SW, Jang HJ, Park SJ, Moon YS, Park JW, Kim CM. Plasma levels of D-dimer and soluble fibrin polymer in patients with hepatocellular carcinoma: a possible predictor of tumor thrombosis. Thromb Res. 2003; 109:125–9. 10.1016/s0049-3848(03)00183-x 12706641

[19] Takeda S, Katoh H, Takaki A, Okamoto K, Ohsato K. Increased fibrin/fibrinogen degradation products without increase of plasmin-alpha 2-plasmin inhibitor complex after hepatectomy for hepatocellular carcinoma. Thromb Res. 1990; 57:289–300. 10.1016/0049-3848(90)90328-a 2156347

[20] Hua N, Chen A, Yang C, Dong H, He X, Ru G, Tong X, Zhou F, Wang S. The correlation of fibrinogen-like protein-1 expression with the progression and prognosis of hepatocellular carcinoma. Mol Biol Rep. 2022; 49:7911–9. 10.1007/s11033-022-07624-6 35776395 PMC9304048

[21] Liu J, Sun G, Pan S, Qin M, Ouyang R, Li Z, Huang J. The Cancer Genome Atlas (TCGA) based m^6^A methylation-related genes predict prognosis in hepatocellular carcinoma. Bioengineered. 2020; 11:759–68. 10.1080/21655979.2020.1787764 32631107 PMC8291839

[22] Barrett T, Wilhite SE, Ledoux P, Evangelista C, Kim IF, Tomashevsky M, Marshall KA, Phillippy KH, Sherman PM, Holko M, Yefanov A, Lee H, Zhang N, et al. NCBI GEO: archive for functional genomics data sets—update. Nucleic Acids Res. 2013; 41:D991–5. 10.1093/nar/gks1193 23193258 PMC3531084

[23] Liberzon A, Birger C, Thorvaldsdóttir H, Ghandi M, Mesirov JP, Tamayo P. The Molecular Signatures Database (MSigDB) hallmark gene set collection. Cell Syst. 2015; 1:417–25. 10.1016/j.cels.2015.12.004 26771021 PMC4707969

[24] Ritchie ME, Phipson B, Wu D, Hu Y, Law CW, Shi W, Smyth GK. limma powers differential expression analyses for RNA-sequencing and microarray studies. Nucleic Acids Res. 2015; 43:e47. 10.1093/nar/gkv007 25605792 PMC4402510

[25] Zhang MY, Huo C, Liu JY, Shi ZE, Zhang WD, Qu JJ, Yue YL, Qu YQ. Identification of a Five Autophagy Subtype-Related Gene Expression Pattern for Improving the Prognosis of Lung Adenocarcinoma. Front Cell Dev Biol. 2021; 9:756911. 10.3389/fcell.2021.756911 34869345 PMC8636677

[26] Chen L, Zhang YH, Lu G, Huang T, Cai YD. Analysis of cancer-related lncRNAs using gene ontology and KEGG pathways. Artif Intell Med. 2017; 76:27–36. 10.1016/j.artmed.2017.02.001 28363286

[27] Niemira M, Collin F, Szalkowska A, Bielska A, Chwialkowska K, Reszec J, Niklinski J, Kwasniewski M, Kretowski A. Molecular Signature of Subtypes of Non-Small-Cell Lung Cancer by Large-Scale Transcriptional Profiling: Identification of Key Modules and Genes by Weighted Gene Co-Expression Network Analysis (WGCNA). Cancers (Basel). 2019; 12:37. 10.3390/cancers12010037 31877723 PMC7017323

[28] Liang JY, Wang DS, Lin HC, Chen XX, Yang H, Zheng Y, Li YH. A Novel Ferroptosis-related Gene Signature for Overall Survival Prediction in Patients with Hepatocellular Carcinoma. Int J Biol Sci. 2020; 16:2430–41. 10.7150/ijbs.45050 32760210 PMC7378635

[29] Zhang Z, Zeng X, Wu Y, Liu Y, Zhang X, Song Z. Cuproptosis-Related Risk Score Predicts Prognosis and Characterizes the Tumor Microenvironment in Hepatocellular Carcinoma. Front Immunol. 2022; 13:925618. 10.3389/fimmu.2022.925618 35898502 PMC9311491

[30] Li S, Que Y, Yang R, He P, Xu S, Hu Y. Construction of Osteosarcoma Diagnosis Model by Random Forest and Artificial Neural Network. J Pers Med. 2023; 13:447. 10.3390/jpm13030447 36983630 PMC10056981

[31] Van Calster B, Wynants L, Verbeek JFM, Verbakel JY, Christodoulou E, Vickers AJ, Roobol MJ, Steyerberg EW. Reporting and Interpreting Decision Curve Analysis: A Guide for Investigators. Eur Urol. 2018; 74:796–804. 10.1016/j.eururo.2018.08.038 30241973 PMC6261531

[32] Yang L, Gu D, Wei J, Yang C, Rao S, Wang W, Chen C, Ding Y, Tian J, Zeng M. A Radiomics Nomogram for Preoperative Prediction of Microvascular Invasion in Hepatocellular Carcinoma. Liver Cancer. 2019; 8:373–86. 10.1159/000494099 31768346 PMC6873064

[33] Subramanian A, Tamayo P, Mootha VK, Mukherjee S, Ebert BL, Gillette MA, Paulovich A, Pomeroy SL, Golub TR, Lander ES, Mesirov JP. Gene set enrichment analysis: a knowledge-based approach for interpreting genome-wide expression profiles. Proc Natl Acad Sci U S A. 2005; 102:15545–50. 10.1073/pnas.0506580102 16199517 PMC1239896

[34] Lei T, Qian H, Lei P, Hu Y. Ferroptosis-related gene signature associates with immunity and predicts prognosis accurately in patients with osteosarcoma. Cancer Sci. 2021; 112:4785–98. 10.1111/cas.15131 34506683 PMC8586685

[35] Ning W, Acharya A, Li S, Schmalz G, Huang S. Identification of Key Pyroptosis-Related Genes and Distinct Pyroptosis-Related Clusters in Periodontitis. Front Immunol. 2022; 13:862049. 10.3389/fimmu.2022.862049 35844512 PMC9281553

[36] Newman AM, Liu CL, Green MR, Gentles AJ, Feng W, Xu Y, Hoang CD, Diehn M, Alizadeh AA. Robust enumeration of cell subsets from tissue expression profiles. Nat Methods. 2015; 12:453–7. 10.1038/nmeth.3337 25822800 PMC4739640

[37] Li T, Fan J, Wang B, Traugh N, Chen Q, Liu JS, Li B, Liu XS. TIMER: A Web Server for Comprehensive Analysis of Tumor-Infiltrating Immune Cells. Cancer Res. 2017; 77:e108–10. 10.1158/0008-5472.CAN-17-0307 29092952 PMC6042652

[38] Thorsson V, Gibbs DL, Brown SD, Wolf D, Bortone DS, Ou Yang TH, Porta-Pardo E, Gao GF, Plaisier CL, Eddy JA, Ziv E, Culhane AC, Paull EO, et al, and Cancer Genome Atlas Research Network. The Immune Landscape of Cancer. Immunity. 2018; 48:812–30.e14. 10.1016/j.immuni.2018.03.023 29628290 PMC5982584

[39] Wang Q, Li M, Yang M, Yang Y, Song F, Zhang W, Li X, Chen K. Analysis of immune-related signatures of lung adenocarcinoma identified two distinct subtypes: implications for immune checkpoint blockade therapy. Aging (Albany NY). 2020; 12:3312–39. 10.18632/aging.102814 32091408 PMC7066911

[40] Wang Z, Wang Y, Yang T, Xing H, Wang Y, Gao L, Guo X, Xing B, Wang Y, Ma W. Machine learning revealed stemness features and a novel stemness-based classification with appealing implications in discriminating the prognosis, immunotherapy and temozolomide responses of 906 glioblastoma patients. Brief Bioinform. 2021; 22:bbab032. 10.1093/bib/bbab032 33839757 PMC8425448

[41] Chen Y, Tang L, Huang W, Abisola FH, Zhang Y, Zhang G, Yao L. Identification of a prognostic cuproptosis-related signature in hepatocellular carcinoma. Biol Direct. 2023; 18:4. 10.1186/s13062-023-00358-w 36750831 PMC9903524

[42] Chen L, Zhou J, Zhao Z, Zhu Y, Xing J, An J, Guo X. Low Expression of Phosphodiesterase 2 (PDE2A) Promotes the Progression by Regulating Mitochondrial Morphology and ATP Content and Predicts Poor Prognosis in Hepatocellular Carcinoma. Cells. 2022; 12:68. 10.3390/cells12010068 36611861 PMC9818237

[43] Kwa MQ, Herum KM, Brakebusch C. Cancer-associated fibroblasts: how do they contribute to metastasis? Clin Exp Metastasis. 2019; 36:71–86. 10.1007/s10585-019-09959-0 30847799

[44] Khorana AA. Cancer and coagulation. Am J Hematol. 2012 (Suppl 1); 87:S82–7. 10.1002/ajh.23143 22389165 PMC3495606

[45] Nadir Y. Decreasing Tumor Growth and Angiogenesis by Inhibition of Coagulation. Semin Thromb Hemost. 2019; 45:622–8. 10.1055/s-0039-1693473 31398734

[46] Chen KD, Wang CC, Tsai MC, Wu CH, Yang HJ, Chen LY, Nakano T, Goto S, Huang KT, Hu TH, Chen CL, Lin CC. Interconnections between autophagy and the coagulation cascade in hepatocellular carcinoma. Cell Death Dis. 2014; 5:e1244. 10.1038/cddis.2014.212 24853422 PMC4047908

[47] Panasiuk A, Zak J, Panasiuk B, Prokopowicz D. Increase in expression of monocytic tissue factor (CD142) with monocytes and blood platelet activation in liver cirrhosis. Blood Coagul Fibrinolysis. 2007; 18:739–44. 10.1097/MBC.0b013e3282ef99f6 17982314

[48] Sakon M, Kambayashi J, Taniguchi K, Yoshida T, Shiba E, Kawasaki T, Tsuji Y, Yukawa M, Murata K, Gotoh M. Effects of transcatheter hepatic arterial embolization on coagulation and fibrinolysis in patients with hepatocellular carcinoma. Am J Gastroenterol. 1991; 86:1800–3. 1660219

[49] Liu Y, Zhang Y, Du D, Gu X, Zhou S. PCDH17 is regulated by methylation of DNMT3B and affects the malignant biological behavior of HCC through EMT. Exp Cell Res. 2022; 418:113245. 10.1016/j.yexcr.2022.113245 35688280

[50] Xiang Y, Yang Y, Lin C, Wu J, Zhang X. MiR-23a-3p promoted G1/S cell cycle transition by targeting protocadherin17 in hepatocellular carcinoma. J Physiol Biochem. 2020; 76:123–34. 10.1007/s13105-020-00726-4 31994011

[51] Alpini G, Glaser SS, Zhang JP, Francis H, Han Y, Gong J, Stokes A, Francis T, Hughart N, Hubble L, Zhuang SM, Meng F. Regulation of placenta growth factor by microRNA-125b in hepatocellular cancer. J Hepatol. 2011; 55:1339–45. 10.1016/j.jhep.2011.04.015 21703189 PMC3184370

[52] Huang Y, Xu J, Xie C, Liao Y, Lin R, Zeng Y, Yu F. A Novel Gene Pair CSTF2/DPE2A Impacts Prognosis and Cell Cycle of Hepatocellular Carcinoma. J Hepatocell Carcinoma. 2023; 10:1639–57. 10.2147/JHC.S413935 37791068 PMC10544262

[53] Xie M, Cai L, Li J, Zhao J, Guo Y, Hou Z, Zhang X, Tian H, Li A, Miao Y. FAM110B Inhibits Non-Small Cell Lung Cancer Cell Proliferation and Invasion Through Inactivating Wnt/β-Catenin Signaling. Onco Targets Ther. 2020; 13:4373–84. 10.2147/OTT.S247491 32547070 PMC7245470

[54] Huang H, Li H, Zhao T, Khan AA, Pan R, Wang S, Wang S, Liu X. TSPAN1-elevated FAM110A promotes pancreatic cancer progression by transcriptionally regulating HIST1H2BK. J Cancer. 2022; 13:906–17. 10.7150/jca.66404 35154458 PMC8824879

[55] Liu Y, Hong W, Zhou C, Jiang Z, Wang G, Wei G, Li X. miR-539 inhibits FSCN1 expression and suppresses hepatocellular carcinoma migration and invasion. Oncol Rep. 2017; 37:2593–602. 10.3892/or.2017.5549 28393215 PMC5428223

[56] Tang JC, Liu JH, Liu XL, Liang X, Cai XJ. Effect of fibulin-5 on adhesion, migration and invasion of hepatocellular carcinoma cells via an integrin-dependent mechanism. World J Gastroenterol. 2015; 21:11127–40. 10.3748/wjg.v21.i39.11127 26494967 PMC4607910

[57] Evan GI, Vousden KH. Proliferation, cell cycle and apoptosis in cancer. Nature. 2001; 411:342–8. 10.1038/35077213 11357141

[58] Currie E, Schulze A, Zechner R, Walther TC, Farese RV Jr. Cellular fatty acid metabolism and cancer. Cell Metab. 2013; 18:153–61. 10.1016/j.cmet.2013.05.017 23791484 PMC3742569

[59] Hu J, Cao J, Topatana W, Juengpanich S, Li S, Zhang B, Shen J, Cai L, Cai X, Chen M. Targeting mutant p53 for cancer therapy: direct and indirect strategies. J Hematol Oncol. 2021; 14:157. 10.1186/s13045-021-01169-0 34583722 PMC8480024

[60] Sangro B, Sarobe P, Hervás-Stubbs S, Melero I. Advances in immunotherapy for hepatocellular carcinoma. Nat Rev Gastroenterol Hepatol. 2021; 18:525–43. 10.1038/s41575-021-00438-0 33850328 PMC8042636

