# Supplementary material for: Establishment and verification of a prognostic model based on coagulation and fibrinolysis-related genes in hepatocellular carcinoma
Source: Aging (Albany NY). 2024 Apr 1;16(9):7578–95. doi: 10.18632/aging.205699 (PMC11131995; doi:10.18632/aging.205699)
Supplement: Supplementary Figures [file aging-16-205699-s001.pdf]

SUPPLEMENTARY FIGURES

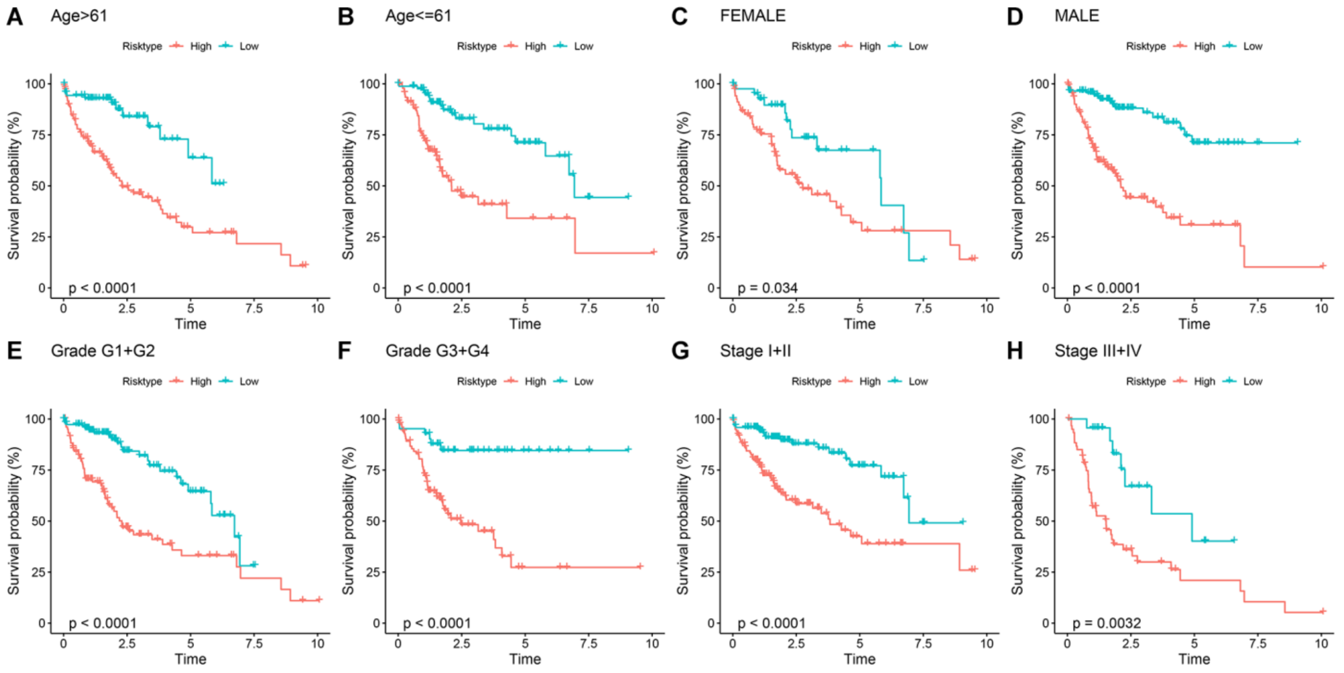

Supplementary Figure 1. Survival state between different RiskScore groups across clinicopathological features (**A**–**H**) of TCGA-HCC cohort.

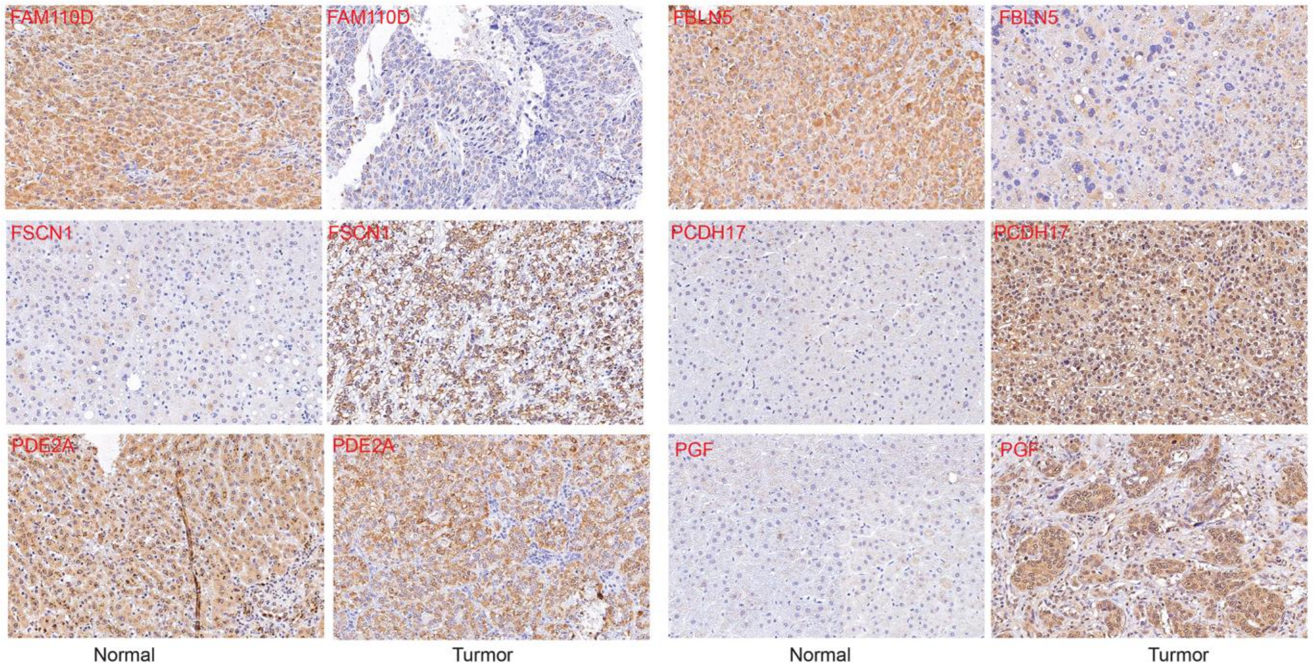

Supplementary Figure 2. Immunohistochemistry validation of key genes.
